# Burnout Related to Diabetes Mellitus: A Critical Analysis

**DOI:** 10.2174/17450179-v18-e2209010

**Published:** 2022-10-21

**Authors:** Konstantinos Kontoangelos, Athanasios Raptis, Vaia Lambadiari, Marina Economou, Sofia Tsiori, Vasiliki Katsi, Christos Papageorgiou, Sofia Martinaki, George Dimitriadis, Charalabos Papageorgiou

**Affiliations:** 1 1^st^ Department of Psychiatry, Eginition Hospital, Medical School National & Kapodistrian University of Athens, Athens, Greece; 2 University Mental Health Neurosciences and Precision Medicine Research Institute “Costas Stefanis”, Athens, Greece; 3 2^nd^ Department of Internal Medicine-Propaedeutic-Research Institute and Diabetes Center, Athens University Medical School, Attikon University Hospital, Athens, Greece; 4 1^st^ Department of Cardiology, “Hippokration” Hospital, Medical School National & Kapodistrian University of Athens, Athens, Greece; 5 National and Kapodistrian University of Athens, Medical School, Athens, Greece

**Keywords:** Burnout, Depersonalization, Diabetes mellitus, Mental health, Depression, Self-care, Parental burnout

## Abstract

**Background:**

Diabetes burnout is a condition when a patient with diabetes feels tired from his/her disease and neglects it for a certain period or continuously.

**Objective:**

Diabetes burnout is frequent, and there is extended literature about psychosocial stress and its negative effects on health.

**Methods:**

A search for relevant studies was conducted using PubMed, Google Scholar and ResearchGate. A systematic review was conducted on the relevant articles after critical appraisal. Only publications in English were selected. The objective of this study was to evaluate the association between burnout syndrome and diabetes mellitus.

**Results:**

This article mainly focused on studies that evaluated the presence of burnout and diabetes mellitus effects. Diabetes can influence psychological health equally with somatic strength. Relatives can also express depression, guilt, fright, worry, rage, and burnout. Psychosocial job stress and extended working hours are linked with a higher possibility of myocardial infarction, diabetes mellitus, and hypertension.

**Conclusion:**

Diabetes burnout is a combination of emotions and practices, ranging from tiredness to indifference, linked with a distressing sense of hopelessness. Revealing this health condition is necessary so that preventive measures can be taken.

## 1 INTRODUCTION

Diabetes is one of the major health challenges of the 21^st^ century. In the United States, it is estimated that about 12% of the adult population has diabetes, and this percentage will probably rise to 28% by 2050 [1]. Diabetes is a condition the patient must manage on his own and negatively influences everyday life, with the psychosocial and emotional burden [2]. Diet, exercise, ordinary medication, and daily blood glucose monitoring are the therapeutic obligations of patients with diabetes mellitus [3]. The patient must always keep his blood sugar levels stable, and if a person does not achieve autonomy in the emotional and physical characteristics, it will affect his health condition [4]. In diabetes mellitus, patients often complain about feelings of distress and frustration, anxiety and depression. Diabetes patients who experience burnout can express symptoms of stress that negatively influence blood sugar levels and lead to non-compliance with managing their therapy [5]. As used by people with diabetes and health care providers, the term diabetes burnout refers to both a psychosocial awareness and a state accompanied by feelings of exhaustion and frustration interconnected to the inflexible daily demands of managing the illness, resulting in contradictory self-care attitudes [6]. This paper aims to review the latest studies on burnout and diabetes mellitus considering the outcomes of diabetes burnout.

## 2 METHODS

An integrative review methodology is adopted as this enables wide inclusion of criteria, incorporating a multiplicity of study methodologies and purposes to capture the depth and breadth of a topic. The literature search was done using the Medline computer database. For six months, an analysis of the specific studies was performed to investigate the relationship between diabetes mellitus and burnout. The search strategy and queries were reviewed and approved by a reference librarian. Strategies were uniquely designed, relative to each database, and integrated controlled vocabulary for databases (for example, MeSH terms for PubMed). Additional sources were identified by checking the reference lists and subsequent citations of all included articles. Data analyses followed established integrative review processes for data reduction, data display, and data comparison. The following terms were selected from titles, abstracts, and keywords: “burnout”, “diabetes mellitus”, and “mental health.” The search focused on burnout patients with diabetes, parents with children with diabetes, and the relationship between diabetes and occupational stress. Only publications in English were selected.

## 3 RESULTS

### 3.1 Burnout of Patients with Diabetes Mellitus

Burnout is a psychological syndrome emerging as a prolonged response to chronic interpersonal stressors on the job. The three key dimensions of this response are overwhelming exhaustion, feelings of cynicism and detachment from the job, and a sense of ineffectiveness and lack of accomplishment. The significance of this three-dimensional model is that it clearly places the individual stress experience within a social context and involves the person’s conception of both self and others. When the construct of burnout was first proposed in the 1970s, there were arguments that it was not a distinctly different phenomenon but rather a new label for an already known state, *i.e*., “old wine in a new bottle.” However, there were a lot of differing opinions about the “already known state”. These included job dissatisfaction, anomie, job stress, anxiety, anger, depression, or some combination of the above. For example, one psychoanalytic perspective was that burnout was not distinguishable from either job stress or depression but represented a failure to achieve narcissistic satisfaction in the pursuit of ideals. Diabetes burnout is a mixture of emotions and behaviors where the patients can feel mentally and physically tired from the daily engagement with self-care and experience unconcern about the illness [7]. Patients with T1DM often feel indifference toward illness characteristics, diabetes self-care, and support systems. Patients often describe levels of diabetes burnout ranging from ‘feeling burned out’ to ‘being burned out’ [8]. Four major themes associated with diabetes burnout are: (i) feeling mentally drained and physically tired of dealing with self-care; (ii) experiencing a disconnection from self, diabetes concern, and support systems; (iii) being impotent and paralyzed to get away from diabetes burnout; (iv) potential contributing factors to diabetes burnout. Self-description of diabetes burnout refers to a combination of emotions and behaviors ranging from exhaustion to detachment accompanied by an intense sense of vulnerability and impotence [9]. In patients (age>18 years) with T1DM, burnout is extensively associated with depression and diabetes agony. While measures of diabetes burnout, distress, and depressive symptoms are important predictors of multiple diabetes outcomes, overall diabetes burnout is no longer significantly related to these outcomes after regulating diabetes distress and depressive symptomatology [10]. Type-2 diabetes patients develop various psychological symptoms that, in combination with other stressors, frequently worsen the stress of diabetes and provoke burnout. It has been observed that improved self-understanding and a greater sense of stability can reduce symptoms of burnout, depression, and anxiety [11]. Age is one of the major determinants of chronic and acute diseases, and their effective treatment can decrease the mortality rate [12]. Psychological stress, which follows experiencing stressful life events, can create a modified immune response and maladaptive immune alterations that can last longer than the event and affect the organism long after the discontinuation of stress. Continuous evaluation of the longitudinal kinetics of peripheral white blood cells in response to stressful life events (SLEs) revealed a change in neutrophils and tended to correlate with increased hair cortisol. It has been observed that SLE activates immunological modifications and stimulates a continuous effect on WBC dispensation. This effect might progress subclinical inflammatory procedures and a correlation among stress, burnout, and physical illness [13]. Compassion fatigue is common among diabetes care providers and is associated with a sense of emotional tiredness that caregivers experience during work. Burnout, as a term, suggests a greater degree of empathy fatigue which involves experts’ activities and personal health and may lead to interception of the caring engagement. A caregiver initially may feel difficult to give support but might become overwhelmed by this obligation later on [14]. Burnout syndrome can cause overwork or loss of interest or the incapacity to understand prospects or to change the system, and the tendency to make work the focus of one’s life [15]. Healthcare professionals and patients can suffer equally from burnout. Stress and disappointment with diabetes management can lead to patients’ burnout. Healthcare professionals should be prepared to deal with this barrier and adjust their attitudes toward the patient accordingly [16]. Prediabetes (preDM) and diabetes, as complex conditions, place considerable tension on medical providers. Moreover, physicians’ awareness regarding patient barriers has a positive association with their probability of prescribing metformin for preDM [17]. A reduced prevalence of prediabetes will occur if the rate decreases or if the median duration of the condition decreases. The duration of prediabetes will tend to minimize as a target of increased screening activity because of the re-classification of individuals to normoglycaemia, reducing the prediabetes rate [18]. The association between psychosocial behavior and diabetes management revealed that participants with emotional burnout due to diabetes-related stress showed poor glycemic control (89.4%) compared to the group with low stress (55.6%) [19]. Diabetes is mainly a self-managed disease with a major psychological impact on the lives of patients and their families. The impact of attitudes and illness beliefs as determinants of patients' health behaviors is essential. Misperceptions concerning the severity and proper management of diabetes can slow down the energetic participation of the patient in the treatment, such as unwillingness to start insulin therapy. Finally, increasing negative experiences can result in a state of “learned helplessness” or “diabetes burnout” [20]. Another point of interest is that there is an important difference between couple burnout, sexual claim ability, and dysfunctional sexual viewpoints in women with diabetic and non-diabetic husbands. Women with non-diabetic husbands have a higher average score in sexual assertiveness compared to women with diabetic husbands, whereas in couple burnout and sexual dysfunctional beliefs factors, women with diabetic husbands have a higher average score [21] (Table **1**).

### 3.2 Burnout in Parents of Children with Diabetes Mellitus

Common themes explored in the Diabetes Online Community (DOC) participation include peer support, advocacy, self-expression, seeking and sharing diabetes information, improving approaches to diabetes data management, and humor. A small number of data exist concerning the impact of DOC involvement on glycemic outcomes, but initial research suggests a positive impact on experiences, attitudes toward diabetes, and promises in diabetes management behaviors [22]. Non-adherent behaviors are usually personal factors, such as awkwardness, low self-efficacy, and diabetes burnout, as well as associated factors, such as insufficient social support and unsatisfactory communication with health care providers [23]. Patients with type 2 diabetes can be tested with a real-time continuous glucose monitor (RT-CGM) as an intervention to improve glycemic control and evaluate glucose response patterns. In a clinical trial, data were collected from the first 12 weeks of a 52-week, prospective, randomized trial comparing RT-CGM while implementing self-monitoring of blood glucose in an agenda with five patterns. The patterns were identified that focused on people with higher starting A1Cs, using it short-term (*e.g*., 2 weeks). Furthermore, it was observed that monitoring for worsening glycemia due to burnout might be the best approach to using RT-CGM in people with type 2 diabetes who do not take prandial insulin [24]. Another important topic is the experience of mothering a child with diabetes when experiencing burnout. In a study, the authors focused on twenty-one mothers of children with T1DM experiencing burnout. It was found that inner feelings derived from an extremely challenging experience of mothering, surrounding involuntary responsibility and stable evaluation [25]. In 21 YouTube videos about diabetes burnout created by parents of children with TIDM, four primary themes were studied related to diabetes burnout in this population: “*exhaustion compounded by stress*,” “*powerlessness to control diabetes*,” “*grief for the loss of a normal life*,” and “*coping strategies*.” The findings helped to highlight how parents of children with T1DM may experience diabetes burnout and inform about diabetes care and the role of social media in improving family center care [26]. Family-based interventions using optimistic support and behavioral contracts, contact skills and problem-solving training, cooperation about diabetes management goals, and parental participation lead to improved daily practices, glycemic control, and family relations. Group interventions for young people with diabetes aiming to improve their stress management skills have shown positive results in medication compliance, thereby improving glycemic control and quality of life [27]. Exploring the effects of group intervention by estimating changes in self-rated clinical burnout and performance-based self-esteem could be an effective option for individual support for parents of chronically ill children with burnout symptomatology [28]. In a study evaluating 252 parents of children with T1DM for both genders, parental burnout was associated with low social support, lack of free time, financial difficulties and an observation that the child’s illness affects each day with a different degree of gravity. Low self-esteem and a strong need for control and surveillance were observed to be the risk factors for parental burnout. Clinicians must be familiar with parents’ behaviors and concerns as well as psychosocial issues and should consider the daily life circumstances and these parents’ psychological support [29]. The fear of the threat of hypoglycemia (FH) is common in parents of young children with T1DM and is associated with poor adjustment behaviors to avoid low blood glucose, parenting stress, and burnout. Forty-three families of young children with T1DM participated in the study, and 36 completed the Reducing Emotional Distress for Childhood Hypoglycemia in Parents (REDCHiP) intervention. Parents attended 94% of intervention sessions, and commitment to the treatment manual was 89%. Parents that reported positive influences of the REDCHiP intervention stated that their understanding was improved in association with fear alertness, coping strategies, behavioral parenting strategies, and support [30]. In the context of parenting a chronically ill child, the prevalence of burnout symptoms was evaluated in a sample of 252 parents of children with TIDM and 38 parents of children with Inflammatory Bowel Disease. The results revealed that considerably more parents of children with chronic diseases (36%) scored for clinical burnout compared to parents of healthy children (20%). Burnout symptoms were more pronounced among mothers of children with diabetes than fathers of children with diabetes. However, mothers and fathers of children with inflammatory bowel disease also reported higher levels of various burnout symptoms [31]. Parental Burnout (PB) is a chronic stress-related disorder and also a severe psychopathological condition with negative consequences for parents and children. Hair cortisol concentration (HCC) is a valid biomarker of a variety of chronic stress situations. In this study, authors compared the HCC levels of parents suffering from PB to that of control parents, and the results showed that HCC was 213% higher in parents suffering from PB compared to control parents. Furthermore, HCC was significantly related to PB. The findings suggested that HCC can be measured as a biomarker of PB and emphasized the view that HCC is a biomarker of persistent stress circumstances [32]. Shared medical appointments (SMAs) in adolescents with diabetes T1DM with multi-component interventions with many medical specialties improve glycemic control, and psychosocial outcomes in poorly controlled adolescent T1DM may be helpful. SMAs focus on self-management, contact skills, target setting, glucose pattern acknowledgment, and peer/diabetes team support. SMAs include individual history and medical history, labs, surveys, multidisciplinary educational ice breakers, and group sessions. The results showed that HbA1c worsened in the 9 months before the study but remained stable during the study. There were significant improvements in overall Quality of Life (QOL), school tasks, psychosocial function, barriers, devotion, and communication. SMAs are feasible replacements for individual appointments in adolescent T1DM, stabilizing glycemic control and improving QOL [33]. A qualitative descriptive analysis of questionnaire free-text comments from children, adolescents and carers participating in DEPICTED identified the emotional impact of living with T1DM and how health professionals' communication skills in the clinic affect the patient/carer experience. Healthcare professionals caring for children/adolescents with T1DM and carers need training in patient-centered communication skills [34]. Nurse educators play an important role in supporting women with gestational diabetes mellitus by providing day instruction and support. The nature of a diabetes educator is difficult and stressful, and facing issues of low literacy increases the workload and the stress in their jobs. Work overload renders diabetes nurse educators vulnerable to burnout, and they may require support to manage their increasing work fatigue. Women with gestational diabetes mellitus must be trained in the management of diabetes to decrease the danger of hyperglycaemia for the fetus. Monitoring the progress of specific learning and educational programs is important [35]. Another important issue is whether the psychological distress and the symptoms of burnout, depression, stress and anxiety in parents of children with chronic conditions can be predictable. The 75 parents of children with chronic conditions having burnout symptoms who participated in an intervention study completed measures of burnout, stress, anxiety, depression, experiential avoidance, cognitive defusion, and mindfulness. The authors confirmed the hypothesis on the importance of psychological flexibility as a central factor in understanding the occurrence of psychological distress [36]. It is important to assess barriers to compliance in adolescent patients in order to improve glycemic control in relation to the psychological factors that may affect compliance. The results of the study showed that the subscales that measure stress, exhaustion and autonomy support are also related to glycemic control [37] (Table **2**).

### 3.3 Burnout, Job Stress, and Diabetes Mellitus

In a study involving 677 participants, a systematic follow-up was conducted on male and female employees for 3 to 5 years regarding the possible occurrence of type 2 diabetes (T2DM). Burnout was assessed by the Shirom-Melamed Burnout Measure with its three subscales: emotional exhaustion, physical fatigue, and cognitive weariness. The results showed that burnout symptoms were remarkably consistent over the follow-up period, irrespective of changes in place of work and employment status, and 17 workers developed T2DM [38]. Similarly, in another study, burnout was associated with a positive effect on diabetes incontinence, stress and the impact of work on health and the conflict between health and work [39]. A qualitative study evaluated the experiences of healthcare providers in caring for people with diabetes in terms of compliance with the emotions associated with treating people with disabilities, fatigue and the work environment. Diabetes-related distress is described as a part of the caregiving practice in health care providers who treat people with diabetes [40]. In a study involving 1554 primary care clinicians in 172 primary care clinics for patients with depression and diabetes and/or cardiovascular disease, the authors found that a substantial minority (31%) experienced burnout that was associated with lower career satisfaction and also lower satisfaction with the remuneration of resources for the treatment of difficult patients. The majority of clinicians thought that a cooperative model of care would be very supportive for treating complex patients [41]. Acquiring medicare patient supplies is an insignificant issue for health professionals who are called upon to explain why a patient needs to be examined more than 3 times a day, and each must complete documents certifying this need. In the United States, many factors contribute to the exhaustion of doctors due to bureaucracy [42]. The relationship between job burnout and work in relation to self-reported treatment for health conditions (cardiovascular condition, high cholesterol, depression, diabetes, hypertension, and irritable bowel syndrome) was investigated while being controlled regarding age, gender, smoking, and alcohol use. The sample included 7895 South African workers. The results showed that burnout was positively associated with self-reported treatment for depression, diabetes, hypertension, and irritable bowel syndrome. The results of this study demonstrated the relationship between burnout, work and treatment for a health condition. There was also evidence of increased treatment reporting for burnout associated with ill conditions [43]. In the Netherlands, a study highlighted the fact that specialist nursing (*e.g*., diabetic nursing) is a current trend in many countries. Moreover, the working status of the specialized nurses is further studied. Comparisons were made between 3 different samples: 1204 nurses employed in 15 hospitals, 1058 nurses in 14 nursing homes and 350 diabetic nurses working in other health care centers throughout the Netherlands. Social support and role conflict scored low for diabetes nurses who perceived both role autonomy and uncertainty. Diabetes nurses also scored the highest in terms of basic work motivation and job happiness and the lowest in psychosomatic potency [44]. Nearly 47% of endocrinologists reported symptoms of burnout, and that number is still increasing. The effects of burnout include personal factors, such as stress, depression and suicide risk, as well as managing consequences, such as reduced quality of care, increased clinical errors, reduced patient empathy, reduced patient satisfaction, and higher pay rates, with some doctors leaving work at the end. Exhaustion affects the organizational level and creates high costs associated with the replacement, recruitment, and retraining of endocrinologists. Endocrinologists considered the feeling of lack of appreciation from management, bureaucracy, and low wages as important factors contributing to exhaustion [45]. In another study, researchers examined the association of work stress with diabetes and prediabetes in a sample of German industrial workers. Diabetes and prediabetes were diagnosed with the glycosylated hemoglobin A1c criterion or the fasting plasma glucose criterion. The overall prevalence rates of diabetes and prediabetes were 3.5 and 42.2%, respectively. The results showed that work-related stress was associated with diabetes. Findings suggest that work-related stress is associated with diabetes and prediabetes in German male industrial workers [46]. In another study, the authors analyzed the association of burnout with high demands, low decision range, and work stress with type 2 diabetes. A low sense of cohesion (SOC) (a factor for successful Stress management) is related to T2DM. A study involving 4,821 healthy Swedes found that low decision range was associated with T2DM. A combination of low SOC and low decision latitude was associated with T2DM [47]. Diabetes alone is not related to fatigue and related health complaints. Fatigue is a symptom of the disease and the mental burden associated with self-management of the disease. Workers with non-comorbid diabetes do not experience more fatigue-related complaints than workers without persistent illnesses. Employees with multiple chronic conditions often report fatigue-related complaints [48]. It has been predicted that the majority of endocrinologists in the future will be women. Although job satisfaction remains constant, exposure rates are high in combination with challenges over the course of a career. The common effects of work-related gender segregation and the gender pay gap are predicted to negatively affect the salaries of endocrinologists of both sexes. The under-representation of women in academic leadership has little impact on endocrinology. Gender biases that are evident in patient satisfaction measures can greatly affect endocrinology [49]. Burnout syndrome is associated with frustration and depressive-like symptoms. This common tendency is parallel to the notion of “disintegration” described by Kernberg with a “black and white” perceptual dichotomy between early idealization and subsequent frustration. One study examined the link between burnout syndrome, depression and Kernberg's idea of ​​splitting 132 women health professionals working with a population of diabetics. The study showed that disintegration as a defense mechanism could be an important step in predicting the symptoms of burnout [50]. The correlation between burnout and metabolic syndrome of female nurses is interesting. In one study, the socio-demographic, occupational, anthropometric (weight, waist circumference, blood pressure) and biochemical (glucose, serum lipids) variables were evaluated. The incidence of burnout and metabolic syndrome of 168 nurses was 19.6% and 38.7%, respectively. It is essential to develop strategies to prevent burnout and metabolic syndrome in nurses, especially those who work at night [51]. Job burnout of physicians was found to be associated with less happiness and a greater willingness to leave the profession. Exhaustion is found to be associated with a lack of time, organization, and control. However, it is not associated with poor quality care or error. The quality of patient care is protected but at a high personal cost for health professionals [52]. The diabetes nurse instructors may be excluded from the hospital; however, they may be the only person providing diabetes education, but this may not be fully recognized by the hospital. It is difficult for a nurse instructor to communicate successfully with the hospital unless there is support from other health professionals. However, loneliness and separation can also result from the nurse instructors’ limited performance space. Although diabetes has many facets, the nursing educator may believe that this focus of nursing practice is highly specialized but needs to be supplemented with additional information [53]. The shift from medical care to the acute management of episodic care to the long-term management of chronic diseases, such as hypertension, diabetes and obesity, gives doctors a sense that they do not offer significant work to patients but leads to a reduced sense of personal achievement. Qualitative analyses for the care of chronic diseases also indirectly encourage doctors to aggressively prescribe many drugs for conditions, such as hypertension and diabetes, with little incentive or reward for the time they devote to counseling, training or helping patients adopt a healthier lifestyle. These difficulties, together with the increasing administrative and secretarial responsibilities, affect the resilience and effectiveness of the physician and contribute to the emotional exhaustion and depersonalization that characterizes burnout [54]. In one study, researchers analyzed the issue of NHS counselors' job well-being and their psychological state in relation to the work environment. An important empirical element of this study is the existence of symptoms of burnout that mediate the observed relationship between job independence and work stress in relation to the symptoms of psychological morbidity of the interviewed counselors. The important role of burnout as a variable is of particular concern given the high prevalence of counselors who are rated as “high” on burnout measures and psychological morbidity symptoms. These results underscore the importance of preventing and reducing burnout and focusing on the progress of attention and autonomy among counselors as an important safeguard plan [55]. People with diabetes will need support from mental health professionals after the end of the pandemic. Many employees working in clinical psychology units have been deployed to support COVID-19 units. Critical work and long-term planning must ensure that diabetes psychologists will be able to return to their original field of work without compromising the availability of COVID-19-specific support services [56]. In an online community of self-identified maternal physicians based in the United States, 16.4% of those surveyed reported regular care or support for people with serious health problems and long-term illnesses.

These maternal physicians had significantly higher rates of depression or anxiety and burnout than other maternal physicians. Female doctors are at high risk for work-family conflict. These results highlight the additional care tasks of some women physicians and the negative impact they have on their day-to-day careers and their behavioral health [57]. Another study found evidence that community health workers (CHW) and federally certified health centers (FQHCs) are building their own property or participating with other community funds to build networks, but they need more transportation and time to explore these networks more effectively and more completely [58]. Furthermore, the literature describes a relationship between workplace injury and the development of diabetes T2DM but without this being associated with aggravating risk factors, such as obesity or lack of exercise. Grouped controlled trials need to be conducted focusing on reducing burnout in workplaces with the goal of randomization to investigate whether stress management and reduction could be a successful means of reducing the risk of T2DM in working populations. Given the potential sample size required for such a test, the most cost-effective way to proceed may be to engage with substitute diabetes risk biomarkers, such as fasting or post-load glucose [59]. An important factor in implementing a team-based service delivery is the requirement for teams to have daily interactions. The care team of doctors, nurses and administrators should gather to comment on their daily schedules, monitor problems, and develop solutions to overcome them. The authors examined whether the impact of a behavioral engagement of leadership training and problem-solving during the daily accumulation process would result in a higher consistency tangle in the intervention arm and result in improved team morale, reduced burnout, and improved outcomes. The authors of this study aimed to provide data on the impact of a behavioral intervention on the application of huddles as a key component of group-based care models [60]. Healthy Work Place (HWP) is a study that proves that work interventions can help clinicians express burnout or even attain professional happiness. HWP interventions focused on the workflow repair drive in communication between vendor teams and chronic disease management programs. Quality development programs may lag behind the clinical life of the clinician while addressing the concerns of clinicians, such as burnout. If burnout is reduced, clinics can focus more effectively on quality-related initiatives [61]. The authors demonstrated that the majority of the medical residents had an acceptable level of stress, and 18% of the medical residents had a high level of stress. Female residents had significantly higher levels of anxiety than male residents, which was detected by multiple regression analysis. Stress was significantly associated with residents of obstetrics and gynecology, surgery, internal medicine and pediatrics in most countries. The specialty of obstetrics and gynecology had the highest rate of burnout among all specialties [62]. SONRISA is a Spanish/English mental health curriculum toolbox developed for promotores (community health workers) who work with Hispanic clients for the prevention and management of diabetes. Promotores and community members from a community-based project requested their university partners to help promotores address depression observed in their clients with diabetes. SONRISA provides training material to address depression and diabetes, educational material for clients, and approaches to prevent work-related emotional burnout [63]. In Iran, the authors studied work stress and burnout in hospital staff in Iran. The rate of work stress and burnout in hospital staff was high on average. Training in problem-solving skills, stress management, budget allocation in healthcare facilities, increasing material and moral incentives for employees on various occasions, setting up sports clubs, providing competent staff and arranging regular meetings between managers and employees can help employees to provide high-quality healthcare services. Work-related stress and burnout can be reduced. Paying attention to staff and managers' preferences can reduce the effects of work-related stress and burnout [64]. High levels of burnout and low levels of productive work, job completion and place of work, as well as social support, are associated with poor behaviors and health outcomes. Exhaustion is significantly associated with diet, physical activity, sleep duration, sleep quality, diabetes, stress, and depression. The value of work, workplace and social support can also be linked to sleep. Sleep can disturb many normal processes and trigger threats of chronic diseases, improving the psychosocial work atmosphere and causing negative effects on public health [65]. Patients with chronic working conditions are more likely to leave work than healthy workers. Interventions and policies to prevent and reduce paid employment should focus mainly on older workers, workers on high sick leave, more burnout complaints, and poor subjective health workers, indicating that their chronic illness limits work or that they receive little support from colleagues. If these supportive interventions and policies give a positive result, the future employment, quality of life, financial situation, and future working capacity of workers with chronic illness can be satisfactorily improved [66]. Difficult patient encounters in the primary care office are common and are associated with physician burnout. A study aimed to determine the effect of difficult encounters on patient health outcomes and the role of physician dissatisfaction and burnout as mediators of this result. Dissatisfaction and burnout were higher among physicians reporting higher frequencies of difficult encounters. Physician perception of frequent difficult encounters was not found to be linked with worse patient care quality or more medical mistakes [67].The transformation experienced by 2 family doctors was compared concerning their practices implemented in the Primary Care Redesign (PCR) team-based model to improve access, quality, and experience without increasing cost. In the pilot practice, compared with reimplementation, there were improvements in total activities and rates of hypertension control, colorectal cancer screening, and most diabetic quality metrics. In the 2^nd^ wave of practices, total appointments increased slightly when clinicians added pre-PCR and then increased substantially after implementation. Also, initial variables, such as hypertension regulation, improved rapidly after implementation. The 2^nd^ wave of practices regarding colorectal cancer screening enhanced gradually, then accelerated post achievement, while diabetic metrics initially remained steady or declined, then improved post accomplishment. Over time, all experiential domains improved for clinicians and most remained stable for staff. Clinician burnout was reduced by at least one-half in both practices except during low staffing periods, which also harmfully affected staff [68] (Table **3**).

## 4 DISCUSSION

In the care of patients with diabetes, the bio-psychosocial approach and solution to problems can be helpful. The ability to understand the patient's point of view will significantly help health professionals respond more effectively and adapt treatment according to the real needs of the patient for the purpose of fast and effective treatment [69]. The psychological pressure from daily dealing with diabetes and also the lack of achievements in treating diabetes can be important factors that can contribute to the exhaustion by diabetes. These factors may be exaggerated due to the lack of an effective support system and the existing perception of accuracy and fear around diabetes and life changes [70]. Concerning parents, the feeling among mothers that they are pressured to provide excellent motherhood is related to how family, relatives, and friends are supportive. The strengthening of a support network is significant to minimize the risk of increasing burnout. Parents of children with diabetes mellitus T1DM have reported that over time, friends and relatives often became sources of further tension, even if they were not supportive at first due to their own panic and fear [71]. Workplace stress models are based primarily on a structural model that correlates chronic work-related stressors with stress responses and effective health planning. Moreover, a new scientific approach and point of view called the “minor event model” has been observed in the literature. This approach focuses more on a work-related stressful event than on chronic work conditions, as well as on the dynamic psychophysiological process over time. Regardless of the current methodological problems and difficulties of this approach, it seems to have value in bridging epidemiological data and practical achievements [72].

Dealing with deep-rooted psychological problems requires more sessions; indeed, more than six-session treatments have had a positive impact on physical function [73], and family caregivers need to receive more information about the patients’ condition to calibrate the communication at their emotional state [74]. It has been observed that Diabetes Mellitus (DM) has a high level of demand on patients, with consistent day-to-day management often required to optimize glycaemic control and minimize complications that may be difficult for autistic individuals [75]. Burnout syndrome is a great psychosocial problem and has been causing concern on the part of researchers and health institutions, owing to the seriousness of its consequences at the individual and organizational levels [76] and can affect physical and mental health, causing sleep pattern alteration, fatigue, concentration deficit, and irritability; on the other hand, it can produce impairment in quality of care and patients’ health outcomes, besides increased absenteeism [77]. It is important to effectively diagnose and prevent diabetes burnout to improve individual-centered diabetes care. In clinical practice, the regular dimension of diabetes anxiety, the exhaustion associated with diabetes care, and the diabetes approach and self-care motivation can help identify individuals at high risk of diabetes burnout. Adequate diagnosis and detection of diabetes burnout, as well as the development of an effective measure of diabetes burnout, will help healthcare providers deal more effectively with the psychosocial issues of diabetes. Imperceptible and chronic physical, mental and social problems occur in diabetes and can become overwhelming and aggravating for both the patient and the doctor.

## CONCLUSION

A realistic and sincere approach leads to the conclusion that for some, life with diabetes is not easy at all. Given that diabetes can require a great deal of personal effort and making rapid decisions every day, it is remarkable that anyone can manage diabetes successfully day after day and even year after year. Classification and treatment of patients and caregivers with burnout could lead them to overcome naivety in diabetes care and bridge the gap between psychological research and the management of diabetes.

## Figures and Tables

**Table 1:** Studies on burnout in patients with diabetes mellitus.

**Author**	**Design**	**Results**
Abdoli S., 2019 [8]	Qualitative study	Detachment may explain poor outcomes in individuals experiencing diabetes burnout.
Lindström C., 2017 [22-25]	Qualitative descriptive study	Diabetes burnout is related to exhaustion to detachment.
Abdoli S., 2020 [9]	Participants completed an online survey including measures of diabetes distress, depressive symptoms, and proposed measures of diabetes burnout	Diabetes burnout is significantly associated with both depression and diabetes distress.
Charman D., 2000 [11]	Psychotherapeutic case presentation	Enhanced self-understanding can help achieve a greater sense of balance and reduce symptoms of burnout.
Nath KA., 2020 [12]	Systematic analysis	Burden of treatment as “the work of being a patient,” and called for minimally disruptive medicine as a model that can mitigate such burden and work.
Penz M., 2018 [13]	Clinical trial	Stressful life events trigger immunological alterations that promote a continuous effect on WBC distribution.
Kalra S., 2020 [14]	Editorial	Living with person with diabetes requires compassion and empathy.
Holyoke A., 1984 [15]	Editorial	Diabetes educators should take pride in their knowledge and performance.
Hoover JW., 1983 [16]	Systematic analysis	When considering patient compliance, healthcare professionals should be aware of this phenomenon as well as their own attitudes and approaches toward the patient.
Seehusen DA., 2018 [17]	Clinical trial	Physician attitudes and subjective norms may predict adherence to guidelines.
Ebrahim S., 2011 [18]	Editor’s choice	Interventions for job strain will reduce coronary heart disease risk.
Roy K., 2020 [19]	Cross-sectional study	Diabetes-related stress, appraisal of diabetes and obesity are the major factors that affect glucose control.
Snoek FJ., 2002 [20]	Systematic analysis	Negative experiences can result in a state of “learned helplessness” or “diabetes burnout.”
Khodabakhshi Koolaee A., 2013 [21]	Clinical trial	There was a significant difference between couple burnout, sexual assertiveness, and sexual dysfunctional beliefs in women with diabetic and non-diabetic husbands.

**Table 2:** Studies on diabetic children’s burnout and their parents.

**Author**	**Design**	**Results**
Hilliard ME., 2015 [22]	Review	The impact of Diabetes Online Community involvement on glycemic outcomes has a positive role in emotional experiences and attitudes toward diabetes.
Pyatak EA., 2017 [23]	Original research	On adherent, behaviors are personal factors, such as embarrassment, low self-efficacy, and diabetes burnout, as well as contextual factors, such as poor social support and poor communication with health care providers.
Fonda SJ., 2013 [24]	Randomized controlled trial	Targeting people with higher starting A1Cs, using it short-term (*e.g*., 2 weeks), and monitoring for worsening glycemia that might be the result of burnout may be the best approach to using RT-CGM in people with type 2 diabetes who do not take prandial insulin.
Lindström C., 2017 [25]	Qualitative study	Mission impossible, an inner feeling derived from an extremely challenging experience of mothering, encompassing involuntary responsibility and constant evaluation.
Abdoli S., 2020 [9]	Qualitative descriptive study	The findings of this study served to clarify how parents of children with T1D may experience diabetes burnout. The findings also demonstrate diabetes care and highlight the role of social media in improving family center support.
Delamater AM., 2018 [27]	Controlled intervention research	Group interventions for young people with diabetes targeting coping and stress management skills have also shown positive effects on regimen adherence, glycemic control, and quality of life.
Lindström C., 2015 [28]	Clinical trial	A group intervention could be an effective alternative to individual support for parents of chronically ill children suffering from burnout.
Lindström C., 2011 [29]	Population-based study	In parents of children with T1DM, clinicians should recognize parents’ attitudes as well as situational psychosocial issues and pay attention to the day-to-day life circumstances in support of these parents.
Marker AM., 2020 [30]	Randomized controlled trial	With REDCHiP intervention, increased knowledge, fear awareness, coping strategies, confidence, behavioral parenting strategies, and support were observed.
Lindström C., 2010 [31]	Study	Burnout may be a useful model for understanding long-term parental responses.
Brianda ME., 2020 [32]	Clinical trial	The HCC levels observed in parents suffering from PB point toward the importance of this condition as well as its potentially harmful consequences for their health.
Floyd BD., 2017 [33]	Clinical trial	SMAs are feasible replacements for individual appointments in adolescent T1DM, improving QOL.
Lowes L., 2015 [34]	Qualitative descriptive analysis	Healthcare professionals caring for children/adolescents\ with T1D and carers need training in patient-centered communication skills.
Carolan M., 2014 [35]	Interpretative phenomenological analysis study	Women with gestational diabetes mellitus GDM are required to master the tasks of diabetes self-management in a very short time to reduce the risk of hyperglycaemia in the fetus.
Sairanen E., 2018 [36]	Intervention study	Cognitive defusion and mindfulness did not make a significant contribution to explaining burnout, stress, and anxiety.
Mulvaney SA., 2011 [37]	Clinical trial	The subscales measuring stress/burnout and autonomy support were also related to glycemic control.

**Table 3:** Studies on burnout, job stress and diabetes mellitus.

**Author**	**Design**	**Results**
Melamed S., 2008 [38]	Prospective study	Burnout symptoms were remarkably consistent over the follow-up period, irrespective of changes in place of work and employment status.
Han TY., 2008 [39]	Cross-sectional design	Job burnout was related to positive effects, level of diabetes (*i.e*., HbA1c), stress from diabetes, and health-to-work conflict.
Craven M., 2019 [40]	Qualitative study	Diabetes-related distress was described as a component of the caregiving experience among healthcare providers who treat PWD.
Whitebird RR., 2017 [41]	Study	The majority of clinicians thought that a collaborative model of care would be very helpful for treating complex patients.
Khoo TK., 2016 [42]	Comment	Numerous factors contribute to physician burnout in the United States, especially the role of bureaucracy and additional non-clinical paperwork.
De Beer LT., 2016 [43]	Cross-sectional Study	Evidence for increased reporting of treatment for ill-health conditions due to burnout was found.
Van den Berg TI., 2008 [44]	Comparative study	Diabetes nursing specialists also scored highest on intrinsic work motivation and job satisfaction and lowest on psychosomatic health.
Gabbay RA., 2020 [45]	Review	Endocrinologists identified feeling a lack of respect from administration, excessive bureaucratic tasks, and insufficient compensation as top contributors to burnout.
Li J., 2013 [46]	Cross-sectional survey	Work stress is associated with diabetes and prediabetes in German industrial male workers.
Agardh EE., 2003 [47]	Cross-sectional study	The combination of low SOC and low decision latitude was associated with type 2 diabetes.
Weijman I., 2004 [48]	Comparative study	Employees with multiple chronic disorders experience more fatigue-related complaints.
Pelley E., 2016 [49]	Systematic analysis	Gender biases evident in patient satisfaction measures, commonly used as proxies for quality of care, may disproportionately impact endocrinology.
Riethof N., 2019 [50]	Study	The defensive mechanism of splitting may allow for the prediction of burnout symptoms, which, in turn, may allow the prediction of burnout syndrome.
Chico-Barba G., 2019 [51]	Observational Study	Associations of emotional exhaustion, personal accomplishment, and night shift with increased waist circumference were found among nurses.
Rabatin J., 2016 [52]	Cross-sectional and longitudinal analyses	Physician burnout was associated with less satisfaction and a greater intent to leave the practice.
H M Jones.,1989 [53]	Study	Diabetes nurse educators may feel that this focus of nursing practice is so specialized that expertise in other areas of practice is lost.
Ha B., 2019 [54]	Special Report	Quality metrics for chronic disease care indirectly encourage physicians to aggressively prescribe multiple medications for conditions, such as hypertension and diabetes.
Khan A., 2018 [55]	Cross-sectional study	The salient role of burnout as an intervening variable is especially concerning given the high prevalence of consultants scoring as ‘high’ on the burnout and psychological morbidity symptom measures.
R. Stewart., 2020 [56]	Commentary	Staff working at the frontline of COVID-19 are provided with adequate psychological care.
Yank V., 2019 [57]	Multicenter study	The findings highlight the additional caregiving responsibilities of some women physicians and the potential consequences of these additional responsibilities for their behavioral health and careers.
Stupplebeen DA., 2019 [58]	Review	Community health workers CHWs engaged in providing community-clinical linkages in multiple grant-specific contexts.
Heikkilä K., 2014 [59]	Meta-analyses.	There is a robust association between job strain and the development of type 2 diabetes irrespective of lifestyle risk factors, such as obesity and physical inactivity.
Branda ME., 2018 [60]	Cluster-randomized trial	There is evidence regarding the impact of a behavioral intervention to implement huddles as a key component of team-based care models.
Linzer M., [61]	Cluster randomized controlled trial	Once burnout is reduced, clinics may then more effectively focus on initiatives related to quality outcomes.
Maswadi N., 2019 [62]	Cross-sectional study	The majority of medical residents had a moderate level of stress, and 18% of the medical residents had a high level of stress.
Reinschmidt KM., 2007 [63]	Review	SONRISA is a Spanish/English mental health curriculum toolbox developed for promotores (community health workers) who work with Hispanic clients to prevent or manage diabetes.
Abarghouei MR., 2016 [64]	Cross-sectional study	The rate of job stress and burnout in hospital personnel is approximately average to high.
Buden JC., 2016 [65]	Cross-sectional observational study	Burnout was significantly associated with nutrition, physical activity, sleep duration, sleep quality, diabetes, and anxiety/depression.
de Boer AGEM., 2018 [66]	Longitudinal cohort study	New interventions and policies to prevent leaving paid employment should especially aim at older employees, employees with high sick leave, and more burnout complaints.
An PG., 2013 [67]	Clinical trial	Dissatisfaction and burnout were higher among physicians reporting higher frequencies of difficult encounters.
Smith PC., 2019 [68]	Clinical trial	Clinician burnout was reduced by at least one-half in both practices except during low staffing periods, which also adversely affected staff.

## LIST OF ABBREVIATIONS

RT-CGM: = Real-Time Continuous Glucose Monitor
REDCHiP: = Reducing Emotional Distress for Childhood Hypoglycemia in Parents
SMAs: = Shared Medical Appointments
QOL: = Quality of Life
T2DM: = Type 2 Diabetes
SOC: = Sense of Cohesion
CHW: = Community Health Workers
DM: = Diabetes Mellitus

## References

[1] Centers for Disease Control and Prevention (2017). National diabetes statistics report.

[2] Lloyd C.E., Brown F.J. (2002). Depression and diabetes.. Curr. Womens Health Rep..

[3] Nelson L.A., Wallston K.A., Kripalani S., LeStourgeon L.M., Williamson S.E., Mayberry L.S. (2018). Assessing barriers to diabetes medication adherence using the Information-Motivation-Behavioral skills model.. Diabetes Res. Clin. Pract..

[4] Beverly E.A., Ivanov N.N., Court A.B., Fredricks T.R. (2017). Is diabetes distress on your radar screen?. J. Fam. Pract..

[5] Polonsky W.H. (1999). Diabetes burnout: what to do when you can’t take it anymore..

[6] Polonsky W.H., Anderson B.J., Rubin R.R. (1996). Practical psychology for diabetes clinicians..

[7] Maslach C., Leiter M.P. (2016). Understanding the burnout experience: recent research and its implications for psychiatry.. World Psychiatry.

[8] Abdoli S., Hessler D., Vora A., Smither B., Stuckey H. (2019). Original research: experiences of diabetes burnout: a qualitative study among people with type 1 diabetes.. Am. J. Nurs..

[9] Abdoli S., Hessler D., Vora A., Smither B., Stuckey H. (2020). Descriptions of diabetes burnout from individuals with Type 1 diabetes: an analysis of YouTube videos.. Diabet. Med..

[10] Abdoli S., Miller-Bains K., Burr E.M., Smither B., Vora A., Hessler D. (2020). Burnout, distress, and depressive symptoms in adults with type 1 diabetes.. J. Diabetes Complications.

[11] Charman D. (2000). Burnout and diabetes: Reflections from working with educators and patients.. J. Clin. Psychol..

[12] Nath K.A. (2020). In the Limelight: March 2020.. Mayo Clin. Proc..

[13] Penz M., Kirschbaum C., Buske-Kirschbaum A., Wekenborg M.K., Miller R. (2018). Stressful life events predict one-year change of leukocyte composition in peripheral blood.. Psychoneuroendocrinology.

[14] Kalra S., Punyani H. (2020). Handling compassion fatigue in diabetes.. J. Pak. Med. Assoc..

[15] Holyoke A. (1984). Editorial.. Diabetes Educ..

[16] Hoover J.W. (1983). Patient burnout, and other reasons for noncompliance.. Diabetes Educ..

[17] Seehusen D.A., Deavers J., Mainous A.G., Ledford C.J.W. (2018). The intersection of physician wellbeing and clinical application of diabetes guidelines.. Patient Educ. Couns..

[18] Ebrahim S. (2011). Hogben on speed, paradoxes and strain.. Int. J. Epidemiol..

[19] Roy K., Iqbal S., Gadag V., Bavington B. (2020). Relationship between psychosocial factors and glucose control in adults with type 2 diabetes.. Can. J. Diabetes.

[20] Snoek F.J. (2002). Breaking the barriers to optimal glycaemic control-what physicians need to know from patients’ perspectives.. Int J Clin.

[21] Khodabakhshi Koolaee A., Asadi E., Mansoor L., Mosalanejad L., Fathabadi A. (2014). A holistic approach to psychological sexual problems in women with diabetic husbands.. Iran. J. Reprod. Med..

[22] Hilliard M., Sparling K., Hitchcock J., Oser T., Hood K. (2015). The emerging diabetes online community.. Curr. Diabetes Rev..

[23] Pyatak B., Florindez D., Weigensberg M.J. (2013). Adherence decision making in the everyday lives of emerging adults with type 1 diabetes.. Patient Prefer. Adherence.

[24] Fonda S.J., Salkind S.J., Walker M.S., Chellappa M., Ehrhardt N., Vigersky R.A. (2013). Heterogeneity of responses to real-time continuous glucose monitoring (RT-CGM) in patients with type 2 diabetes and its implications for application.. Diabetes Care.

[25] Lindström C., Åman J., Norberg A.L., Forssberg M., Anderzén-Carlsson A. (2017). “Mission Impossible”; the mothering of a child with type 1 diabetes - from the perspective of mothers experiencing burnout.. J. Pediatr. Nurs..

[26] Abdoli S., Vora A., Smither B., Roach A.D., Vora A.C. (2020). I don’t have the choice to burnout; experiences of parents of children with type 1 diabetes.. Appl. Nurs. Res..

[27] Delamater A.M., de Wit M., McDarby V., Malik J.A., Hilliard M.E., Northam E., Acerini C.L. (2018). ISPAD Clinical Practice Consensus Guidelines 2018: Psychological care of children and adolescents with type 1 diabetes.. Pediatr. Diabetes.

[28] Lindström C., Åman J., Anderzén-Carlsson A., Lindahl Norberg A. (2016). Group intervention for burnout in parents of chronically ill children - a small-scale study.. Scand. J. Caring Sci..

[29] Lindström C., Åman J., Norberg A.L. (2011). Parental burnout in relation to sociodemographic, psychosocial and personality factors as well as disease duration and glycaemic control in children with Type 1 diabetes mellitus.. Acta Paediatr..

[30] Marker A.M., Monzon A.D., Nelson E.L., Clements M.A., Patton S.R. (2020). An intervention to reduce hypoglycemia fear in parents of young kids with type 1 diabetes through video-based telemedicine (REDCHiP): trial design feasibility, and acceptability.. Diabetes Technol. Ther..

[31] Lindström C., Åman J., Norberg A.L. (2010). Increased prevalence of burnout symptoms in parents of chronically ill children.. Acta Paediatr..

[32] Brianda M.E., Roskam I., Mikolajczak M. (2020). Hair cortisol concentration as a biomarker of parental burnout.. Psychoneuroendocrinology.

[33] Floyd B.D., Block J.M., Buckingham B.B., Ly T., Foster N., Wright R., Mueller C.L., Hood K.K., Shah A.C. (2017). Stabilization of glycemic control and improved quality of life using a shared medical appointment model in adolescents with type 1 diabetes in suboptimal control.. Pediatr. Diabetes.

[34] Lowes L., Eddy D., Channon S. (2015). DEPICTED study team. The experience of living with type 1 diabetes and attending clinic from the perception of children, adolescents and careers: analysis of qualitative data from the DEPICTED study.. J. Pediatr. Nurs..

[35] Carolan M. (2014). Diabetes nurse educators’ experiences of providing care for women, with gestational diabetes mellitus, from disadvantaged backgrounds.. J. Clin. Nurs..

[36] Sairanen E, Lappalainen P, Hiltunen A (2018). Psychological inflexibility explains distress in parents whose children have chronic conditions.. PLoS One.

[37] Mulvaney S.A., Hood K.K., Schlundt D.G., Osborn C.Y., Johnson K.B., Rothman R.L., Wallston K.A. (2011). Development and initial validation of the barriers to diabetes adherence measure for adolescents.. Diabetes Res. Clin. Pract..

[38] Melamed S., Shirom A., Toker S., Shapira I. (2006). Burnout and risk of type 2 diabetes: a prospective study of apparently healthy employed persons.. Psychosom. Med..

[39] Han T. (2008). A biopsychosocial perspective to the burnout of Korean workers with diabetes.. Am. J. Health Behav..

[40] Craven M., Simons Z., de Groot M. (2019). Diabetes distress among healthcare providers: A qualitative study.. Diabetes Res. Clin. Pract..

[41] Whitebird R.R., Solberg L.I., Crain A.L., Rossom R.C., Beck A., Neely C., Dreskin M., Coleman K.J. (2017). Clinician burnout and satisfaction with resources in caring for complex patients.. Gen. Hosp. Psychiatry.

[42] Khoo T.K. (2016). Factors Affecting Burnout in Physicians.. Mayo Clin. Proc..

[43] de Beer L.T., Pienaar J., Rothmann S. (2016). Job burnout, work engagement and self-reported treatment for health conditions in South Africa.. Stress Health.

[44] van den Berg T.I.J., Vrijhoef H.J.M., Tummers G., Landeweerd J.A., van Merode G.G. (2008). The work setting of diabetes nursing specialists in the Netherlands: A questionnaire survey.. Int. J. Nurs. Stud..

[45] Gabbay R.A., Barrett A.M. (2020). Endocrinologist burnout: we need to tackle it and bring joy to work.. J. Clin. Endocrinol. Metab..

[46] Li J., Jarczok M.N., Loerbroks A., Schöllgen I., Siegrist J., Bosch J.A., Wilson M.G., Mauss D., Fischer J.E. (2013). Work stress is associated with diabetes and prediabetes: cross-sectional results from the MIPH Industrial Cohort Studies.. Int. J. Behav. Med..

[47] Agardh E.E., Ahlbom A., Andersson T., Efendic S., Grill V., Hallqvist J., Norman A., Östenson C.G. (2003). Work stress and low sense of coherence is associated with type 2 diabetes in middle-aged Swedish women.. Diabetes Care.

[48] Weijman I., Kant I.J., Swaen G.M., Ros W.J.G., Rutten G.E.H.M., Schaufeli W.B., Schabracq M.J., Winnubst J.A.M. (2004). Diabetes, employment and fatigue-related complaints: a comparison between diabetic employees, “healthy” employees, and employees with other chronic diseases.. J. Occup. Environ. Med..

[49] Pelley E., Danoff A., Cooper D.S., Becker C. (2016). Female physicians and the future of endocrinology.. J. Clin. Endocrinol. Metab..

[50] Riethof N., Bob P., Laker M., Varakova K., Jiraskova T., Raboch J. (2019). Burnout syndrome, mental splitting and depression in female health care professionals.. Med. Sci. Monit..

[51] Chico-Barba G., Jiménez-Limas K., Sánchez-Jiménez B., Sámano R., Rodríguez-Ventura A.L., Castillo-Pérez R., Tolentino M. (2019). Burnout and metabolic syndrome in female nurses: an observational study.. Int. J. Environ. Res. Public Health.

[52] Rabatin J., Williams E., Baier Manwell L., Schwartz M.D., Brown R.L., Linzer M. (2016). Predictors and outcomes of burnout in primary care physicians.. J. Prim. Care Community Health.

[53] Jones H. (1989). A diabetes nurse educator’s view of the hospital and burnout.. Diabetes Educ..

[54] Ha B. (2019). The power of plants: is a whole-foods, plant-based diet the answer to health, health care, and physician wellness?. Perm. J..

[55] Khan A, Teoh KR, Islam S (2018). Psychosocial work characteristics, burnout, psychological morbidity symptoms and early retirement intentions: a cross-sectional study of NHS consultants in the UK.. BMJ Open.

[56] Stewart R. (2020). How do we recover from COVID‐19? Helping diabetes teams foresee and prepare for the psychological harms.. Diabet. Med..

[57] Yank V., Rennels C., Linos E., Choo E.K., Jagsi R., Mangurian C. (2019). Behavioral health and burnout among physician mothers who care for a person with a serious health problem, long-term illness, or disability.. JAMA Intern. Med..

[58] Stupplebeen D.A., Sentell T.L., Pirkle C.M., Juan B., Barnett-Sherrill A.T., Humphry J.W., Yoshimura S.R., Kiernan J., Hartz C.P., Keliikoa L.B. (2019). Community health workers in action: community-clinical linkages for diabetes prevention and hypertension management at 3 community health centers.. Hawaii J. Med. Public Health.

[59] Nyberg S.T., Fransson E.I., Heikkilä K., Ahola K., Alfredsson L., Bjorner J.B., Borritz M., Burr H., Dragano N., Goldberg M., Hamer M., Jokela M., Knutsson A., Koskenvuo M., Koskinen A., Kouvonen A., Leineweber C., Madsen H., Magnusson Hanson L.L., Marmot M.G., Nielsen M.L., Nordin M., Oksanen T., Pejtersen J.H., Pentti J., Rugulies R., Salo P., Siegrist J., Steptoe A., Suominen S., Theorell T., Väänänen A., Vahtera J., Virtanen M., Westerholm P.J.M., Westerlund H., Zins M., Batty G.D., Brunner E.J., Ferrie J.E., Singh-Manoux A., Kivimäki M., IPD-Work Consortium (2014). Job strain as a risk factor for type 2 diabetes: a pooled analysis of 124,808 men and women.. Diabetes Care.

[60] Branda M.E., Chandrasekaran A., Tumerman M.D., Shah N.D., Ward P., Staats B.R., Lewis T.M., Olson D.K., Giblon R., Lampman M.A., Rushlow D.R. (2018). Optimizing huddle engagement through leadership and problem-solving within primary care: A study protocol for a cluster randomized trial.. Trials.

[61] Linzer M., Poplau S., Brown R., Grossman E., Varkey A., Yale S., Williams E.S., Hicks L., Wallock J., Kohnhorst D., Barbouche M. (2017). Do work condition interventions affect quality and errors in primary care? results from the healthy work place study.. J. Gen. Intern. Med..

[62] Maswadi N., Khader Y.S., Abu Slaih A. (2019). Perceived stress among resident doctors in Jordanian teaching hospitals: cross-sectional study.. JMIR Public Health Surveill..

[63] Reinschmidt K.M., Chong J. (2007). SONRISA: a curriculum toolbox for promotores to address mental health and diabetes.. Prev. Chronic Dis..

[64] Abarghouei M.R., Sorbi M.H., Abarghouei M., Bidaki R., Yazdanpoor S. (2016). A study of job stress and burnout and related factors in the hospital personnel of Iran.. Electron. Physician.

[65] Buden J.C., Dugan A.G., Namazi S., Huedo-Medina T.B., Cherniack M.G., Faghri P.D. (2016). Work characteristics as predictors of correctional supervisors’ health Outcomes.. J. Occup. Environ. Med..

[66] de Boer A.G.E.M., Geuskens G.A., Bültmann U., Boot C.R.L., Wind H., Koppes L.L.J., Frings-Dresen M.H.W. (2018). Employment status transitions in employees with and without chronic disease in the Netherlands.. Int. J. Public Health.

[67] An P.G., Manwell L.B., Williams E.S., Laiteerapong N., Brown R.L., Rabatin J.S., Schwartz M.D., Lally P.J., Linzer M. (2013). Does a higher frequency of difficult patient encounters lead to lower quality care?. J. Fam. Pract..

[68] Smith P.C., Lyon C., English A.F., Conry C. (2019). Practice Transformation Under the University of Colorado’s Primary Care Redesign Model.. Ann. Fam. Med..

[69] Fisher L., Polonsky W.H., Hessler D.M., Masharani U., Blumer I., Peters A.L., Strycker L.A., Bowyer V. (2015). Understanding the sources of diabetes distress in adults with type 1 diabetes.. J. Diabetes Complications.

[70] Wennick A., Lundqvist A., Hallström I. (2009). Everyday experience of families three years after diagnosis of type 1 diabetes in children: a research paper.. J. Pediatr. Nurs..

[71] Iwata N. (1997). Strategies for evaluation of stressors at work and their meaning in stress science: 2. A historical view of occupational stress models and stressor measurements.. Job Stress Research.

[72] Kawakami N., Haratani T. (1999). Epidemiology of job stress and health in Japan: review of current evidence and future direction.. Ind. Health.

[73] Rouhi S., Dadkhah P., Firoozi M., Hashemi M. (2020). New Model for Couple Therapy for Patients with Chronic Pain and their Caregivers: An Attempt to Improve Quality of Life and Reduce Pain.. Clin. Pract. Epidemiol. Ment. Health.

[74] Picardi A, Miniotti M, Leombruni P, Gigantesco A. (2021). A qualitative study regarding COVID-19 inpatient family caregivers’ need for supportive care.. Clinical Practice & Epidemiology in Mental Health.

[75] Tromans S., Yao G., Alexander R., Mukaetova-Ladinska E., Kiani R., Al-Uzri M., Chester V., Carr R., Morgan Z., Vounzoulaki E., Brugha T. (2020). The Prevalence of Diabetes in Autistic Persons: A Systematic Review.. Clin. Pract. Epidemiol. Ment. Health.

[76] de Oliveira S.M., de Alcantara Sousa L.V., Vieira Gadelha M.S., do Nascimento V.B. (2019). Prevention Actions of Burnout Syndrome in Nurses: An Integrating Literature Review.. Clin. Pract. Epidemiol. Ment. Health.

[77] Carmassi C., Bertelloni C.A., Avella M.T., Cremone I., Massimetti E., Corsi M., Dell’Osso L. (2020). PTSD and Burnout are Related to Lifetime Mood Spectrum in Emergency Healthcare Operator.. Clin. Pract. Epidemiol. Ment. Health.

